# Prevalence of sexual dysfunction in men with chronic prostatitis/chronic pelvic pain syndrome: a meta-analysis

**DOI:** 10.1007/s00345-015-1720-3

**Published:** 2015-11-06

**Authors:** Hong-Jun Li, De-Ying Kang

**Affiliations:** Urological Department of Peking Union Medical College Hospital, Peking Union Medical College, Chinese Academy of Medical Sciences, Beijing, 100730 China; Department of Evidence-based Medicine and Clinical Epidemiology, West China Hospital, Sichuan University, No. 37 Guo Xue Xiang, Chengdu, 610041 China

**Keywords:** Chronic prostatitis, Chronic pelvic pain syndrome, Prevalence, Sexual dysfunction, Erectile dysfunction, Premature ejaculation

## Abstract

**Purpose:**

This study aims to estimate the prevalence of sexual dysfunction in men with chronic prostatitis/chronic pelvic pain syndrome (CP/CPPS) by conducting a meta-analysis.

**Methods:**

Relevant publications were searched using PubMed, Embase, CBM, China National Knowledge Infrastructure, VIP and Wanfang databases up to August 2015. Studies that reported the prevalence of erectile dysfunction, premature ejaculation and total sexual dysfunction in men with CP/CPPS were included.

**Results:**

A total of 24 studies involving 11,189 men were included. Overall prevalence of sexual dysfunction in men with CP/CPPS was 0.62 (95 % CI 0.48–0.75), while the prevalence of erectile dysfunction and premature ejaculation was 0.29 (95 % CI 0.24–0.33) and 0.40 (95 % CI 0.30–0.50), respectively. From 1999 to 2010, the prevalence of sexual dysfunction, erectile dysfunction and premature ejaculation was 0.65 (95 % CI 0.45–0.83), 0.27 (95 % CI 0.22–0.33) and 0.41 (95 % CI 0.27–0.55), respectively. From 2011 to 2014, the prevalence of sexual dysfunction, erectile dysfunction and premature ejaculation was 0.50 (95 % CI 0.22–0.75), 0.35 (95 % CI 0.29– 0.40) and 0.39 (95 % CI 0.37–0.41), respectively.

**Conclusion:**

The prevalence of sexual dysfunction in men with CP/CPPS was high, even though overall sexual dysfunction demonstrated a slightly decreasing trend. Furthermore, erectile dysfunction prevalence rate had an increasing trend in recent years. More prospective studies are needed to evaluate sexual dysfunction improvement with better management of CP/CPPS.

**Electronic supplementary material:**

The online version of this article (doi:10.1007/s00345-015-1720-3) contains supplementary material, which is available to authorized users.

## Introduction

Prostatitis is classified as acute bacterial prostatitis (category I), chronic bacterial prostatitis (category II), chronic prostatitis (CP)/chronic pelvic pain syndrome (CPPS, category III) and asymptomatic inflammatory prostatitis (category IV), according to the National Institutes of Health (NIH) prostatitis classification system [1]. CP/CPPS, a very common urologic problem, has trivial and complicated symptoms that severely impact the quality of life of patients. Given that monotherapies are usually less effective for alleviating symptoms [2], the UPOINT clinical phenotypic classification system, which has placed major concerns on diversified symptoms, has been established to addresses this puzzle. In this classification system, the phenotype of patients with CP/CPPS is classified into six clinical domains: urinary, psychosocial, organ-specific, infection, neurologic/systemic and tenderness of muscles [3]. The classification and specific treatment of men with CP/CPPS using the UPOINT system have greatly improved the symptoms of CP/CPPS. However, the UPOINT classification does not consider sexual dysfunction, and the inclusion of a sexual dysfunction domain to the UPOINT system is continuously being debated [4].

Sexual dysfunction is one of the distressing health problems that affect men with active sexual activity. The impact of sexual dysfunction on the quality of life of male patients has been well established, and improving sexual dysfunction might help attenuate CP/CPPS symptoms. The relationship between CP/CPPS and sexual dysfunction has often been overlooked [5]. Compared with the general population, men with CP/CPPS appear more likely to experience sexual dysfunction including erectile dysfunction, premature ejaculation, painful ejaculation and decreased sexual desire [6, 7]. However, a wide range of prevalence estimates for sexual dysfunction has been documented in a multitude of independent studies [8]. The reported prevalence of sexual dysfunction varied considerably mainly due to the definitions and methodologies in sexual function studies. Moreover, the development of sexual dysfunction in patients with CP/CPPS is positively linked with the duration of the disease [9, 10].

Several epidemiological studies have investigated the prevalence of sexual dysfunction in men with CP/CPPS. At present, there has been no large epidemiological study to estimate the prevalence of sexual dysfunction in men with CP/CPPS. Estimating the prevalence of sexual dysfunction in men with CP/CPPS would help to better understand morbidity. Therefore, this meta-analysis was aimed to estimate the prevalence of sexual dysfunction in men with CP/CPPS based on the all available studies.

## Materials and methods

### Search strategy

This meta-analysis was conducted according to the Meta-analysis Of Observational Studies in Epidemiology (MOOSE) guidelines [11]. We comprehensively searched for relevant studies using Medline (PubMed), Embase (OVID), CBM, China National Knowledge Infrastructure, VIP and Wanfang databases from the inception of this study to August 2015. The following search keywords with various combinations were used: prevalence OR frequency OR questionnaire OR survey AND sexual dysfunction OR erectile dysfunction OR premature ejaculation AND chronic prostatitis OR chronic pelvic pain syndrome. We also hand-searched the reference lists of included articles to identify additional studies.

### Study selection

Studies that met the following inclusion criteria were included: (1) any type of observational, cohort or cross-sectional study, and case series; (2) original research written in English and Chinese; (3) studies that provide the prevalence of sexual dysfunction in men with CP/CPPS or the total number and the number of sexual dysfunction participants; and (4) studies that at least reported the prevalence of overall sexual dysfunction, erectile dysfunction or premature ejaculation. Erectile dysfunction is defined as the inability to obtain or maintain an erection sufficient for adequate sexual performance. Subjects that scored 21 or less on the International Index of Erectile Function (IIEF) were defined as having erectile dysfunction. Erectile dysfunction, premature ejaculation, decreased sexual desire, ejaculatory pain and so on were summarized as sexual dysfunction. Studies were excluded when patients were limited to a particular type of CP/CPPS or the study was an editorial, review or abstract. If there were several articles of the same population, we selected only papers with the most detailed data.

### Data extraction and quality assessment

Two authors (DY Kang and HJ Li) independently extracted the following information: first author’s surname, publication year, country of data collection, age range of patients, sample size, criterion tools of sexual dysfunction, and prevalence of sexual dysfunction, erectile dysfunction or premature ejaculation. Any disagreements were resolved by discussion between the two reviewers. Authors were contacted when additional data were required.

The reporting quality assessment of the articles included in this review was processed by two independent reviewers (DY Kang and HJ Li) after the data collection process. The reporting bias of included studies was based on the STROBE score [12], which is a methodological checklist that provides 22 key criteria relevant to qualitative research studies. The main items include the article’s title and abstract, introduction, methods, results, discussion sections and other information.

### Statistical analysis

The summary statistic for each individual study was the prevalence proportion and was calculated as the rate of the number of men with sexual dysfunction to the sample size of the studied population. The prevalence and its standard errors were calculated using a standard formula, and then pooled effect size with its corresponding 95 % confidence interval (CI). Heterogeneity across studies was determined by *I*^2^ statistic (significance level of *I*^2^ > 50 %) and Cochran’s Q statistic (*P* < 0.10 was considered statistically significant). In this study, a meta-analysis was conducted to pool prevalence estimates using a random effect model due to anticipated clinical heterogeneity. Based on the wide application of the NIH classification of prostatitis, we conducted subgroup analyses according to publication year (1999–2010 vs. 2011–2014). In addition, subgroup analyses were also performed by study locations (China vs. other areas), criterion tools and sample sizes (≥500 vs. <500). Begg’s rank correlation test [13] and Egger’s regression asymmetry test [14] were used to examine possible publication bias. All statistical analyses were conducted using Stata software version 11.0 (Stata Corp LP, College Station, USA).

## Results

### Literature search

**Fig. 1:**
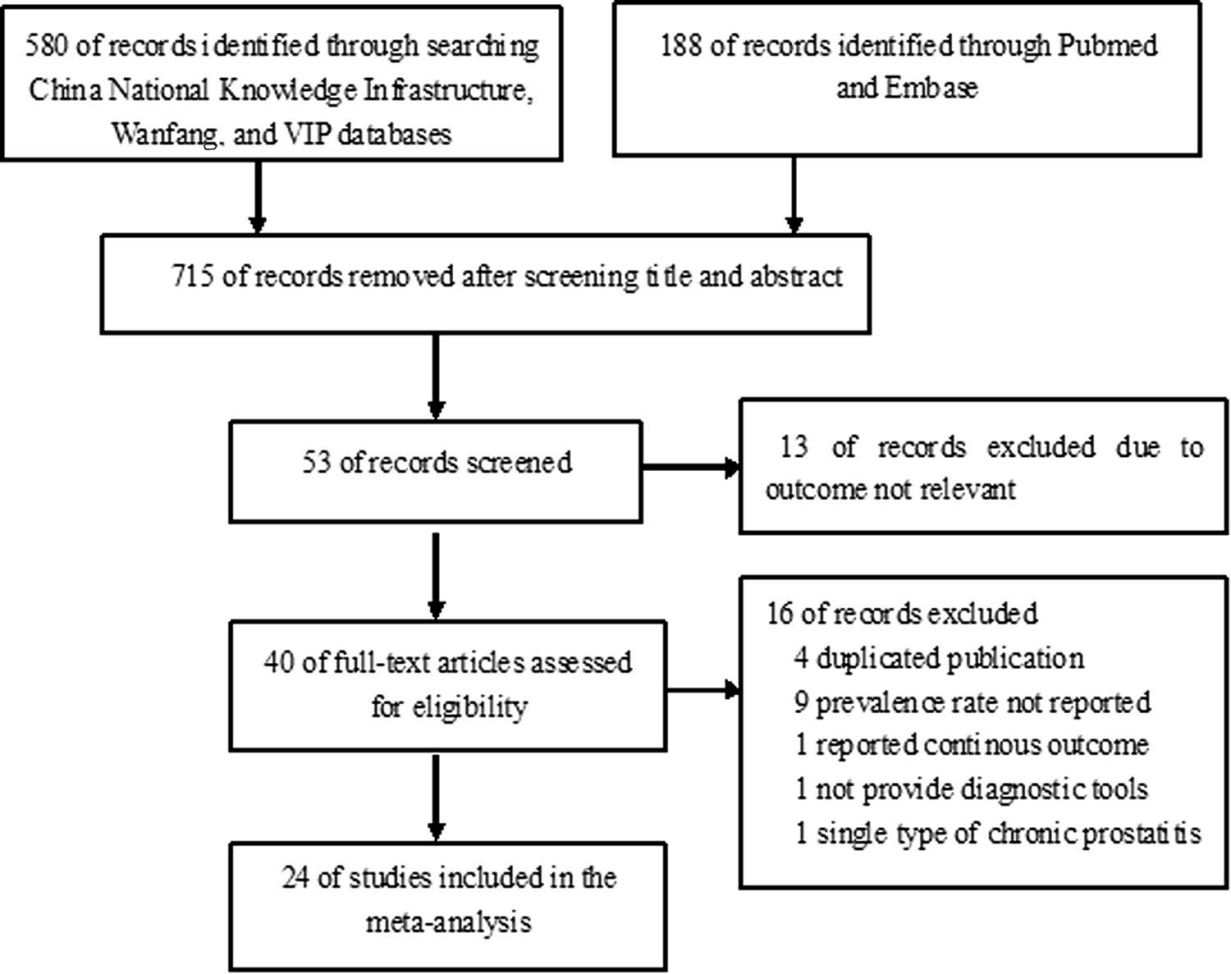
Flowchart of the trial selection process for the meta-analysis

The electronic database search yielded a total of 768 records. After removal of duplicates and scanning by title and abstract, 715 papers were removed. Thus, 53 potentially relevant citations were retrieved for detailed full-text evaluation. Finally, a total of 24 full-manuscript papers [6, 7, 10, 15–35] satisfied the inclusion criteria. The flowchart for the detailed selection process is presented in Fig. 1.

### Study characteristics

A total of 11,189 men with CP/CPPS were included in the current meta-analysis. Seventeen studies (70.8 %) were conducted in China, and the remaining seven studies (29.2 %) were conducted in other areas. All studies were published from 1999 to 2014. Sample sizes ranged from 43 to 2498. Most studies were cross-sectional in design. General characteristics of the 24 studies are presented in Table 1. The overall reporting quality of the studies was moderate to good, and the range of STROBE scores was within 14–19. Risk of reporting bias for individual studies based on STROBE scores is shown in supplemental Table S1.

**Table 1:** Characteristics of the included studies

References	Year	Country	Sample size	Age (years)	Criterion tools	Prevalence of sexual dysfunction	Prevalence of ED	Prevalence of PE
Liang et al. [11]	1999	China	120	29–46	NIH-CPSI + IIEF-5	33.3 %		
Yang et al. [12]	2002	China	500	18–47	NIH-CPSI + CPSFI	81 %	25 %	7 %
Chen et al. [13]	2002	China	160	23–43	NIH-CPSI + IIEF-5	92.5 %		
Hao et al. [14]	2005	China	2498	20–59	NIH-CPSI + IIEF-5	38 %	28.3 %	
Gonen et al. [15]	2005	Turkey	66	21–55	NIH-CPSI			77.3 %
Chen [16]	2006	China	220	>20	NIH-CPSI + IIEF-5	44.5 %	15.5 %	20.9 %
Xv et al. [17]	2006	China	432	22–45	NIH-CPSI + IIEF-5		25.2 %	
Li et al. [18]	2006	China	1000	19–50	NIH-CPSI + IIEF-5 + CISFPE		15.8 %	40.2 %
Qiu et al. [19]	2007	China	623	18–57	CISFPE + IIEF-5		16.9 %	39 %
Anderson et al. [20]	2006	USA	145	18–77	NIH-CPSI + PPSS	92 %		
Trinchieri et al. [21]	2007	Italy	399	<50	NIH-CPSI		34 %	55 %
Bartoletti et al. [22]	2007	Italy	764	25–50	NIH-CPSI + IIEF-5	45.5 %	27.5 %	8.4 %
Lee et al. [4]	2008	Malaysia	296	20–69	NIH-CPSI + IIEF-5	72.3 %	48.3 %	
Lu et al. [23]	2008	China	374	18–65	NIH-CPSI + IIEF-5	88.9 %	55.7 %	72.8 %
Chen et al. [24]	2009	China	198	20–59	NIH-CPSI + IIEF-5		20.7 %	
Lan et al. [25]	2009	China	637	25–61	CISFPE + IIEF-5		17.6 %	28.4 %
Hao et al. [26]	2011	China	370	15–60	NIH-CPSI + IIEF-5		35.1 %	
Chen et al. [7]	2011	China	160	28–52	NIH-CPSI	37.5 %		
Sonmez et al. [3]	2011	Turkey	43	22–48	NIH-CPSI + IIEF	41.86 %	23.25 %	
Hou et al. [27]	2012	China	233	18–62	CISFPE			41.6 %
Wang et al. [28]	2013	China	147	18–64	NIH-CPSI + IIEF-5		45.8 %	
Chen et al. [29]	2013	China	152	20–54	NIH-CPSI + IIEF-5		26.63 %	41.45 %
Zhang et al. [30]	2013	China	1335	14–68	NIH-CPSI + IIEF-5	69.7 %	37.5 %	37.8 %
Cai et al. [31]	2014	Italy	317	33.8 ± 5.1	NIH-CPSI + PEDT			37.2 %

*CPSFI* Chronic Prostatitis-Related Sexual Function Index, *CISFPE* Chinese Index of Sex Function of Premature Ejaculation, *IIEF*-*5* International Index of Erectile Function 5, *PPSS* Pelvic Pain Symptom Survey, *PEDT* Premature Ejaculation Diagnostic Tool

### Overall prevalence of sexual dysfunction

**Fig. 2:**
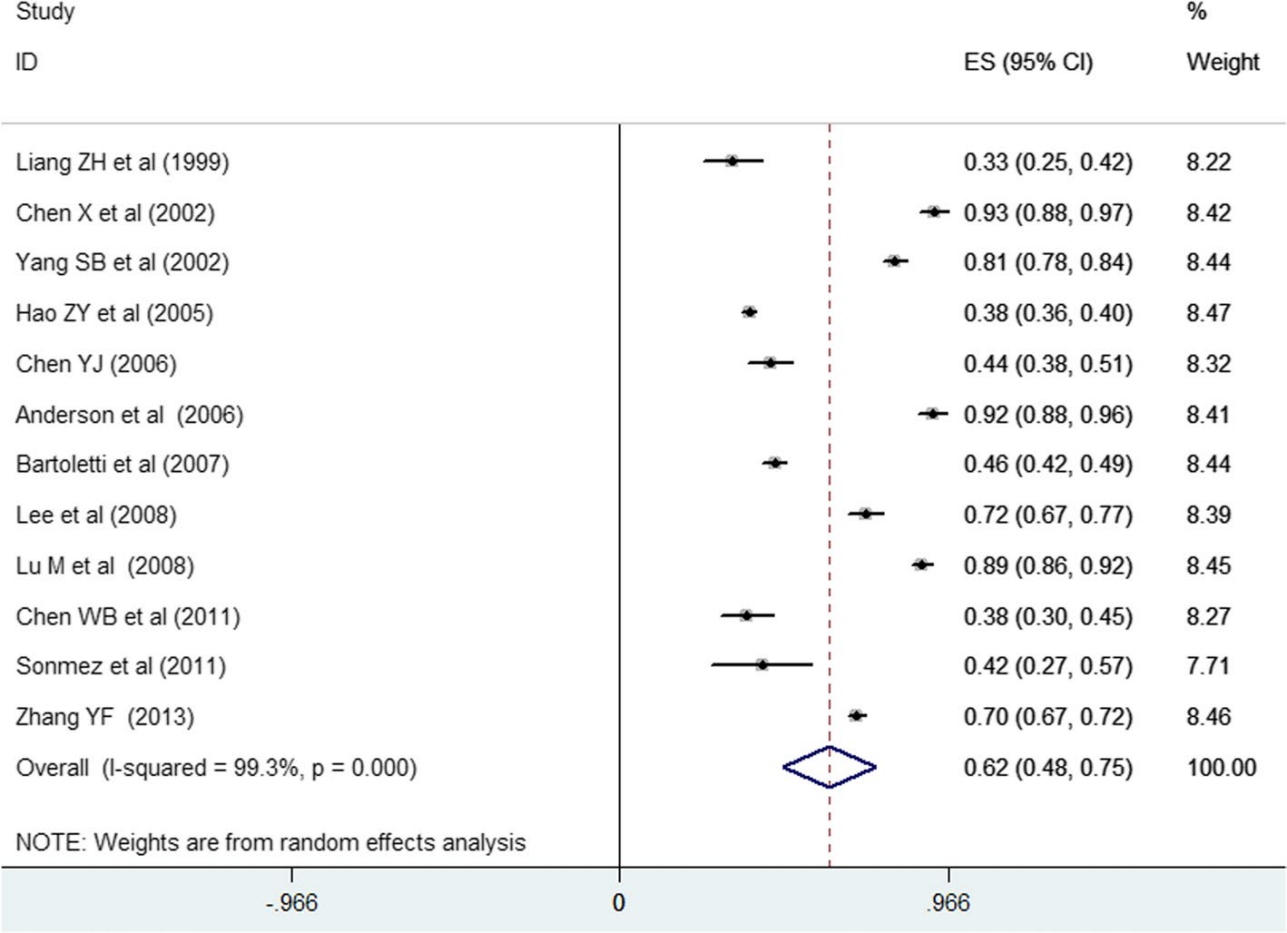
Overall prevalence of sexual dysfunction among men with chronic prostatitis/chronic pelvic pain syndrome in a random effect model

Twelve studies [6, 7, 10, 15–18, 20, 24, 26, 27, 34] involving 6615 patients reported the prevalence of total sexual dysfunction. As shown in Fig. 2, pooled estimation for the prevalence of overall sexual dysfunction among men with CP/CPPS was 0.62 (95 % CI 0.48–0.75) in a random effect model, and significant heterogeneity was observed (*I*^2^ = 99.3 %; *P* < 0.001). Evidences of publication bias were not noted in both the Begg’s rank correlation test (*P* = 0.193) and Egger’s linear regression test (*P* = 0.157).

### Prevalence of erectile dysfunction and premature ejaculation

**Fig. 3:**
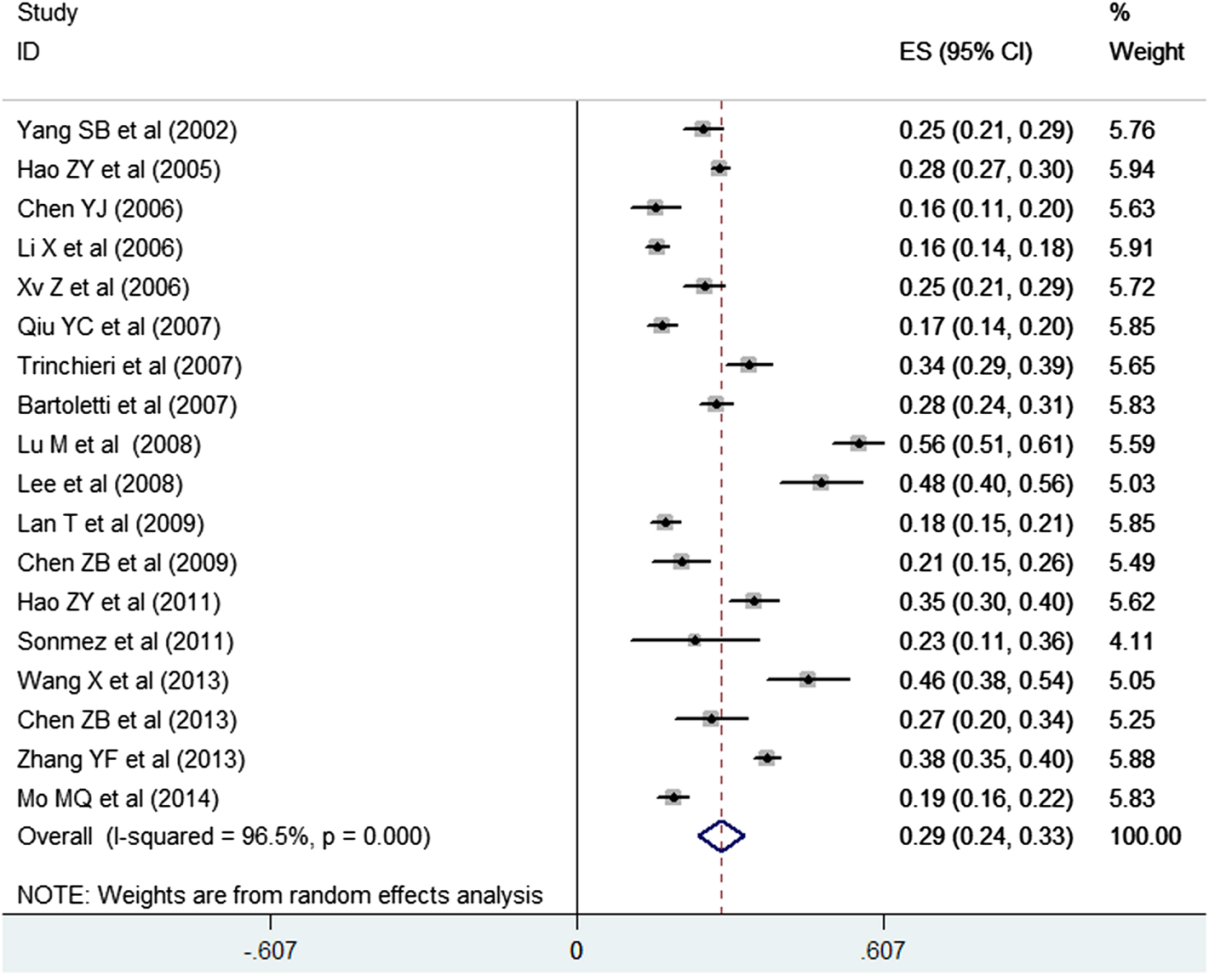
Prevalence of erectile dysfunction among men with chronic prostatitis/chronic pelvic pain syndrome in a random effect model

Seventeen studies [6, 7, 16, 18, 20–23, 25–30, 32–34] involving 9835 patients reported the prevalence of erectile dysfunction. As shown in Fig. 3, pooled estimation for the prevalence of erectile dysfunction among men with CP/CPPS was 0.29 (95 % CI 0.24–0.33; *I*^2^ = 96.5 %; *P* < 0.001) in a random effect model. Both Begg’s rank correlation test (*P* = 0.434) and Egger’s linear regression test (*P* = 0.173) did not reveal evidences of publication bias.

**Fig. 4:**
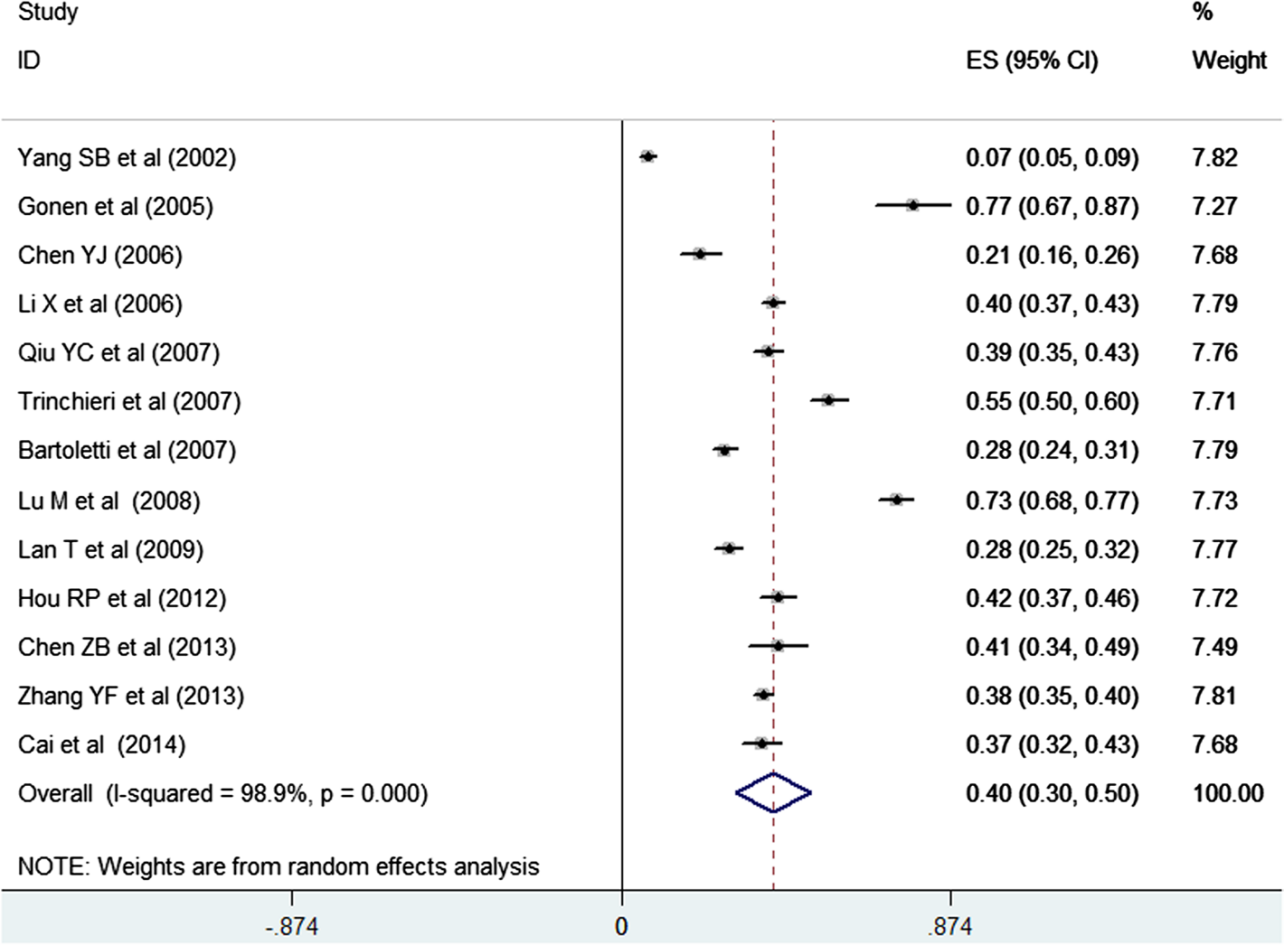
Prevalence of premature ejaculation among men with chronic prostatitis/chronic pelvic pain syndrome in a random effect model

Thirteen studies [16, 19, 20, 22, 23, 25–27, 29, 31, 33–35] involving 6819 patients reported premature ejaculation data. As shown in Fig. 4, pooled estimation for the prevalence of premature ejaculation among men with CP/CPPS was 0.40 (95 % CI 0.30–0.50; *I*^2^ = 98.9 %; *P* < 0.001) in a random effect model. Evidences of publication bias were observed on Egger’s linear regression test (*P* = 0.048), but not in Begg’s rank correlation test (*P* = 0.300).

### Subgroup analyses

From 1999 to 2010, the prevalence of total sexual dysfunction, erectile dysfunction and premature ejaculation was 0.65 (95 % CI 0.48–0.83), 0.27 (95 % CI 0.22–0.33) and 0.41 (95 % CI 0.27–0.55), respectively. From 2011 to 2014, the prevalence of total sexual dysfunction, erectile dysfunction and premature ejaculation was 0.50 (95 % CI 0.25–0.75), 0.35 (95 % CI 0.29–0.40) and 0.39 (95 % CI 0.37–0.41), respectively. In addition, prevalence rates of erectile dysfunction and premature ejaculation were higher in other areas than in China (Table 2).

**Table 2:** Subgroup analyses the prevalence of sexual dysfunction

Subgroup	No. of studies	Pooled effect sizes	95 % confidence interval	Heterogeneity between studies
*Overall prevalence of sexual dysfunction*				
Publication year				
<2011	9	0.65	0.45–0.83	*P* < 0.001; *I* ^2^ = 99.5 %
≥2011	3	0.50	0.25–0.75	*P* < 0.001; *I* ^2^ = 97.3 %
Region				
China	8	0.61	0.43–0.78	*P* < 0.001; *I* ^2^ = 99.5 %
Other areas	4	0.63	0.33–0.88	*P* < 0.001; *I* ^2^ = 98.9 %
Sample size				
<500	8	0.63	0.48–0.79	*P* < 0.001; *I* ^2^ = 98.5 %
≥500	4	0.59	0.38–0.79	*P* < 0.001; *I* ^2^ = 99.6 %
*Prevalence of erectile dysfunction*				
Publication year				
<2011	12	0.27	0.22–0.33	*P* = 0.122; *I* ^2^ = 96.7 %
≥2011	5	0.35	0.29–0.40	*P* = 0.001; *I* ^2^ = 77.5 %
Region				
China	13	0.28	0.22–0.34	*P* < 0.001; *I* ^2^ = 97.1 %
Other areas	4	0.34	0.25–0.42	*P* < 0.001; *I* ^2^ = 87.9 %
Sample size				
<500	10	0.33	0.25–0.41	*P* < 0.001; *I* ^2^ = 95.3 %
≥500	7	0.24	0.18–0.30	*P* < 0.001; *I* ^2^ = 97.3 %
*Prevalence of premature ejaculation*				
Publication year				
<2011	9	0.41	0.27–0.55	*P* < 0.001; *I* ^2^ = 99.2 %
≥2011	4	0.39	0.37–0.41	*P* < 0.001; *I* ^2^ = 0 %
Region				
China	9	0.37	0.24–0.49	*P* < 0.001; *I* ^2^ = 99.1 %
Other areas	4	0.49	0.31–0.67	*P* < 0.001; *I* ^2^ = 98 %
Sample size				
<500	7	0.49	0.35–0.64	*P* < 0.001; *I* ^2^ = 97.9 %
≥500	7	0.30	0.18–0.42	*P* < 0.001; *I* ^2^ = 99.0 %

## Discussion

To the best of our knowledge, this meta-analysis is the first to estimate the prevalence of sexual dysfunction in men with CP/CPPS. This study provides evidence that the overall prevalence of sexual dysfunction among men with CP/CPPS was 62 %. The prevalence of erectile dysfunction and premature ejaculation was 29 % and 40 %, respectively. Specifically, the prevalence of erectile dysfunction among men with CP/CPPS had an increasing trend in recent years.

Stratified analysis by geographical area revealed that the prevalence of erectile dysfunction and premature ejaculation appeared to be low in China than in Western nations. However, these findings should be interpreted with caution due to the limited number of studies conducted in Western nations. Furthermore, overall prevalence of sexual dysfunction among men with CP/CPPS demonstrated a slight decreasing trend in recent years. Subgroup analyses have indicated that the pooled prevalence of erectile dysfunction in men with CP/CPPS increased from 27 % in 1999–2010 to 35 % in 2011–2014, suggesting that the prevalence rate of erectile dysfunction might experience an increase in recent years. The prevalence of premature ejaculation in men with CP/CPPS exhibited a slight decreasing trend, from 41 % in 1999–2010 to 39 % in 2011–2014.

Data on the impact of CP/CPPS on sexual function varied across studies. Sexual dysfunction is highly prevalent in men with CP/CPPS compared with the general population. A cross-sectional study from Singapore indicated that men with CP/CPPS had the worse erectile function as measured with the IIEF assessment tool, compared with men without prostatitis [36].

A case–control study conducted in Taiwan revealed that men with erectile dysfunction were more likely to have had a previous diagnosis of CP/CPPS (OR 3.62; 95 % CI 3.07–4.26) after adjusting for covariates than controls [37].

Recognition of the high prevalence of sexual dysfunction in men with CP/CPPS led to the proposal of adding a sexual domain to the UPOINT system and that the UPOINT plus sexual dysfunction (UPOINTS) typing system for prostatitis may be reasonable. However, the validity of adding a sexual dysfunction domain to the UPOINT system continuous to be debated. Some studies have shown that the inclusion of a sexual domain to the UPOINT system improved its correlation with symptom severity [38, 39] or the quality of life of patients [40], while other studies have indicated that adding a sexual domain did not appear to add value [41]. These conflicting results might be explained by the diverse ethnic and cultural backgrounds of the studied populations. Our study demonstrates that the prevalence of sexual dysfunction among men with CP/CPPS was up to 62 %, and this finding highlights the importance of assessing the sexual domain in men with CP/CPPS. Regular ejaculation is one of the effective methods of treating CP/CPPS. Delaying ejaculation, sexual abstinence and coitus interrupts are all risk factors for CP/CPPS [42]. Therefore, adding a sexual domain to the UPOINT system may develop a UPOINT typing system that could help to differentiate more homogenous UPOINT subgroups and guide individualized therapy.

The underlying mechanisms of CP/CPPS-associated sexual dysfunction remain unclear. Vasculogenic, endocrine and neurogenic factors, as well as psychological factors, may play an important role in the pathogenesis of sexual dysfunction in CP/CPPS. Patients with CP/CPPS are more likely to have nitric oxide-mediated vascular endothelial dysfunction compared to asymptomatic controls, which contribute to sexual dysfunction in these populations [43]. Prostatic calcifications were significantly associated with the presence of erectile dysfunction in males with CP/CPPS [44]. A link between sexual function and chronic prostatitis might be a psychological factor [45]. Sexual dysfunction due to psychological causes in patients with CP/CPPS was high, and men with CP/CPPS experienced more depression and impaired sexual function [46].

Several limitations of this study should be addressed. First, we were unable to analyze the association between the severity of CP/CPPS and sexual dysfunction, because these data were unavailable in most of the studies. Second, the relationship between CP/CPPS and sexual dysfunction might be changed when patients undergo CP/CPPS treatment. In addition, diabetes, coronary artery disease and peripheral vascular disease can all contribute to sexual dysfunction. All above factors were not specifically investigated; thus, the selection bias of patients could not be excluded. Third, substantial heterogeneity across included studies was observed. Subgroup analyses revealed that publication year, sample size or geographical area could not sufficiently explain the significant heterogeneity. We could not account for the sources of heterogeneity, and the diagnostic criteria for sexual dysfunction, heterogeneous diagnosis of NIH prostatitis type I to IV, study design, duration of prostatitis, and age difference of participants might be sources of heterogeneity. Fourth, the age of participants could exert an important impact on the prevalence of sexual dysfunction, particularly on erectile dysfunction. However, subgroup analysis based on age was not performed due to insufficient data in the original articles. Finally, considering the disagreement on quality criteria for assessing cross-sectional studies, the reporting quality of the included studies evaluated using the STROBE score should be interpreted with caution.

## Conclusion

This meta-analysis reveals that the prevalence of sexual dysfunction in men with CP/CPPS is high to some extent. This finding reveals that adding a sexual domain to the UPOINT typing system may be warranted. Considering the limitations noted above, more prospective studies are needed to evaluate sexual dysfunction improvement with better management of CP/CPPS.

## Electronic supplementary material

Below is the link to the electronic supplementary material.

Supplementary material 1 (DOC 131 kb)
