# Supplementary material for: Prevalence of sexual dysfunction in men with chronic prostatitis/chronic pelvic pain syndrome: a meta-analysis
Source: World J Urol. 2015 Nov 6;34:1009–17. doi: 10.1007/s00345-015-1720-3 (PMC4921105; doi:10.1007/s00345-015-1720-3)
Supplement: Supplementary file 1 — Supplementary material 1 (DOC 131 kb) [file 345_2015_1720_MOESM1_ESM.doc]

Supplemental Table S1 Risk bias of the included studies

| **Study/year** | **STROBE Items** | | | | | | | | | | | | | | | | | | | | | | Total scores |
| --- | --- | --- | --- | --- | --- | --- | --- | --- | --- | --- | --- | --- | --- | --- | --- | --- | --- | --- | --- | --- | --- | --- | --- |
|  | (1) | (2) | (3) | (4) | (5) | (6) | (7) | (8) | (9) | (10) | (11) | (12) | (13) | (14) | (15) | (16) | (17) | (18) | (19) | (20) | (21) | (22) |  |
| Liang ZH et al [15] | Yes | Yes | Yes | No | Yes | Yes | Yes | Yes | Yes | Yes | Yes | No | Yes | Yes | Yes | No | Yes | Yes | No | Yes | Yes | No | 17 |
| Yang SB et al [16] | Yes | Yes | Yes | Yes | Yes | Yes | Yes | Yes | Yes | Yes | Yes | No | Yes | Yes | Yes | No | No | Yes | Yes | Yes | Yes | No | 17 |
| Chen X et al [17] | Yes | Yes | Yes | No | Yes | Yes | Yes | Yes | Yes | Yes | No | No | Yes | Yes | Yes | No | No | Yes | Yes | Yes | No | No | 15 |
| Hao ZY et al [18] | No | Yes | Yes | No | Yes | Yes | Yes | Yes | Yes | Yes | No | No | Yes | Yes | Yes | No | No | Yes | Yes | Yes | No | No | 14 |
| Gonen et al [19] | Yes | Yes | Yes | No | Yes | Yes | Yes | Yes | Yes | Yes | Yes | No | Yes | Yes | Yes | Yes | No | Yes | No | Yes | No | No | 16 |
| Chen YJ [20] | Yes | Yes | Yes | No | Yes | Yes | Yes | Yes | Yes | Yes | Yes | No | Yes | Yes | Yes | Yes | No | Yes | No | Yes | No | No | 16 |
| Xv Z et al [21] | Yes | Yes | Yes | Yes | Yes | Yes | Yes | Yes | Yes | Yes | Yes | No | Yes | Yes | Yes | No | No | Yes | No | Yes | No | Yes | 17 |
| Li X et al [22] | Yes | Yes | Yes | Yes | Yes | Yes | Yes | Yes | Yes | Yes | Yes | No | Yes | Yes | Yes | No | No | Yes | Yes | Yes | Yes | No | 17 |
| Qiu YC et al [23] | No | Yes | Yes | No | Yes | Yes | Yes | Yes | Yes | Yes | No | No | Yes | Yes | Yes | No | No | Yes | Yes | Yes | No | No | 14 |
| Anderson et al [24] | Yes | Yes | Yes | Yes | Yes | Yes | Yes | Yes | Yes | Yes | No | No | Yes | Yes | Yes | No | Yes | Yes | Yes | Yes | No | No | 17 |
| Trinchieri et al [25] | Yes | Yes | Yes | Yes | Yes | Yes | Yes | Yes | No | No | Yes | No | Yes | Yes | Yes | No | Yes | Yes | No | Yes | No | Yes | 16 |
| Bartoletti et al [26] | Yes | Yes | Yes | No | Yes | Yes | Yes | Yes | Yes | Yes | No | No | Yes | Yes | Yes | No | Yes | Yes | Yes | Yes | No | No | 16 |
| Lee et al [7] | Yes | Yes | Yes | No | Yes | Yes | Yes | Yes | Yes | Yes | Yes | Yes | Yes | Yes | Yes | No | No | Yes | Yes | Yes | Yes | No | 18 |
| Lu M et al [27] | Yes | Yes | Yes | No | Yes | Yes | Yes | Yes | Yes | Yes | Yes | Yes | Yes | Yes | Yes | No | Yes | Yes | No | Yes | Yes | No | 18 |
| Chen ZB et al [28] | Yes | Yes | Yes | Yes | Yes | Yes | Yes | Yes | Yes | Yes | Yes | No | Yes | Yes | Yes | No | Yes | Yes | Yes | Yes | No | Yes | 19 |
| Lan T et al [29] | Yes | Yes | No | No | Yes | Yes | Yes | Yes | Yes | Yes | No | No | Yes | Yes | Yes | No | Yes | Yes | Yes | Yes | Yes | No | 16 |
| Hao ZY et al [30] | Yes | Yes | Yes | Yes | Yes | Yes | Yes | Yes | Yes | Yes | Yes | No | Yes | Yes | Yes | Yes | Yes | Yes | No | Yes | No | No | 17 |
| Chen WB et al [10] | Yes | Yes | Yes | No | Yes | Yes | Yes | Yes | No | Yes | Yes | No | Yes | Yes | Yes | No | Yes | Yes | No | Yes | No | Yes | 16 |
| Sonmez et al [6] | Yes | Yes | Yes | Yes | Yes | Yes | Yes | Yes | No | Yes | Yes | No | Yes | Yes | Yes | No | No | Yes | No | Yes | No | Yes | 16 |
| Hou RP et al [31] | Yes | Yes | Yes | No | Yes | Yes | Yes | Yes | Yes | Yes | Yes | No | Yes | Yes | Yes | No | Yes | Yes | Yes | Yes | Yes | No | 18 |
| Wang X et al [32] | Yes | Yes | Yes | Yes | Yes | Yes | Yes | Yes | Yes | Yes | Yes | No | Yes | Yes | Yes | No | Yes | Yes | No | Yes | No | No | 16 |
| Chen ZB et al [33] | Yes | Yes | Yes | Yes | Yes | Yes | Yes | Yes | No | Yes | Yes | No | Yes | Yes | Yes | No | Yes | Yes | No | Yes | No | No | 16 |
| Zhang YF et al [34] | Yes | Yes | Yes | Yes | Yes | Yes | Yes | Yes | No | Yes | Yes | No | Yes | Yes | Yes | No | No | Yes | No | Yes | No | Yes | 16 |
| Cai et al [35] | Yes | Yes | Yes | Yes | Yes | Yes | Yes | Yes | No | Yes | Yes | No | Yes | Yes | Yes | No | Yes | Yes | Yes | Yes | Yes | No | 18 |

(1)Title and abstract informative and balanced; (2) Background/rationale stated in the introduction; (3) Objective specified in the introduction; (4) Study design correctly and presented early in the paper; (5) Setting, locations, and relevant dates described; (6) Give the eligibility criteria, and the sources and methods of selection of participants; (7) Diagnostic criteria, outcomes, exposures, predictors, potential confounders, and effect modifiers for all variables clearly defined; (8) Sources of data and details of methods of measurement given for each variable of interest; (9)Any efforts to address potential sources of bias described; (10) How the study size was arrived at clearly explained;(11) Describe all statistical methods, including those used to control for confounding; (12) Describe analytical methods taking account of sampling strategy; (13) Numbers of individuals of study reported; (14) Characteristics of study, number of participants clearly described;(15) Report numbers of outcome events or summary measures; (16) Confounder-adjusted risk estimates and their 95% CI reported; (17) Analyses of subgroups and interactions reported; (18) Summarize key results with reference to study objectives;(19) Discuss limitations of the study;(20) Give a cautious overall interpretation of results;(21) Discuss the generalizability (external validity) of the study results; (22)Source of funding and role of the funders described.
